# Exploiting the Indole Scaffold to Design Compounds Binding to Different Pharmacological Targets †

**DOI:** 10.3390/molecules25102331

**Published:** 2020-05-16

**Authors:** Sabrina Taliani, Federico Da Settimo, Claudia Martini, Sonia Laneri, Ettore Novellino, Giovanni Greco

**Affiliations:** 1 Department of Pharmacy, University of Pisa, Via Bonanno Pisano, 6, 56126 Pisa, Italy; federico.dasettimo@unipi.it (F.D.S.); claudia.martini@unipi.it (C.M.); 2 Department of Pharmacy, University of Naples “Federico II”, Via D. Montesano, 49, 80131 Naples, Italy; sonia.laneri@unina.it (S.L.); ettore.novellino@unina.it (E.N.)

**Keywords:** type A γ-aminobutyric acid (GABA_A_) chloride channel, translocator protein (TSPO), murine double Minute 2 (MDM2) protein, A_2B_ adenosine receptor (A_2B_ AR), Kelch-like ECH-associated protein 1 (Keap1)

## Abstract

Several indole derivatives have been disclosed by our research groups that have been collaborating for nearly 25 years. The results of our investigations led to a variety of molecules binding selectively to different pharmacological targets, specifically the type A γ-aminobutyric acid (GABA_A_) chloride channel, the translocator protein (TSPO), the murine double minute 2 (MDM2) protein, the A_2B_ adenosine receptor (A_2B_ AR) and the Kelch-like ECH-associated protein 1 (Keap1). Herein, we describe how these works were conceived and carried out thanks to the versatility of indole nucleus to be exploited in the design and synthesis of drug-like molecules.

## 1. Introduction

During the last 25 years our research groups have been engaged in preparing and testing several indole derivatives as ligands for some pharmacologically relevant targets: namely, the type A γ-aminobutyric acid chloride channel, the translocator protein, the murine double minute 2 protein, the A_2B_ adenosine receptor and the Kelch-like ECH-associated protein 1. We chose indole as a privileged scaffold owing to its recognized ability of being exploited to obtain drug-like molecules [1]. In virtue of its chemical reactivity, this heterocycle is amenable to be readily modified in order to introduce multiple decorations, so to obtain a multitude of indole-based compounds. Indole is widely distributed among biologically active molecules, either natural (many alkaloids, tryptophan, plant hormones) and synthetic, acting on a huge number of therapeutic targets [1]. The present review summarizes our studies taking indole as the polar star of our medicinal chemistry strategies.

## 2. Indole Derivatives as Ligands of the Benzodiazepine Receptor

**Figure 1:**
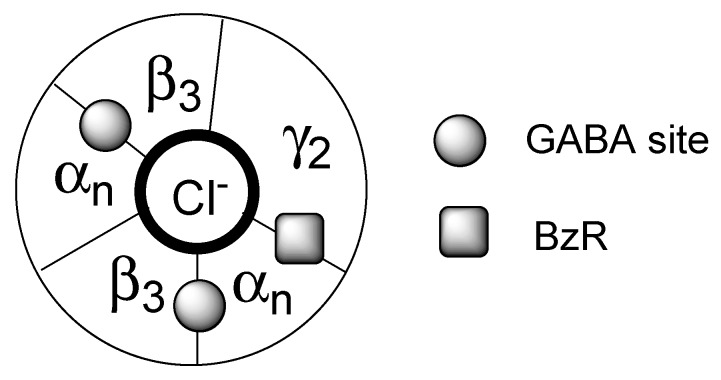
Schematic representation of the organization of the five protein subunits composing the major GABA_A_ complex isoforms. There are two β_3_ subunits, one γ_2_ subunits and two among six α subunits (where n varies from 1 to 6). The circle in bold corresponds to the chloride channel. The binding sites of the neurotransmitter GABA and of the BzR ligands are evidenced by filled circles and, respectively, a square.

Many drugs structurally related to diazepam bind to a site known for a long time as the benzodiazepine receptor (BzR). Although this term was changed in 1998 to “benzodiazepine binding site” [2] the acronym BzR has been widely used for decades and it is still usually employed by the scientific community. This binding site is located within the transmembrane type A γ-aminobutyric acid (GABA_A_) chloride channel, one of the most important members of the family of pentameric ligand-gated ion channels [2]. When this channel is activated by interaction with the neurotransmitter GABA, the flow of chloride into the cell increases and produces hyperpolarisation. Mammals express a number of GABA_A_ isoforms localized in the CNS which are composed by five subunits: two β_3_, one γ_2_ and two α among six types (α_1_, α_2_, α_3_, α_4_, α_5_, α_6_). Such a pentameric organization (Figure 1) originates the following six GABA_A_ subtypes: α_1_β_3_γ_2_, α_2_β_3_γ_2_, α_3_β_3_γ_2_, α_4_β_3_γ_2_, α_5_β_3_γ_2_ and α_6_β_3_γ_2_ [3,4,5]. The above subtypes can be also named by specifying the α subunit which imparts specific physiological and pharmacological properties to the GABA_A_ complex [6,7,8,9,10]. The α_1_ subtype, the dominant one, is present in both the cortex and cerebellum and mediates sedation; the α_2_ and α_3_ subtypes are found mainly in the cortex and the hippocampus and are involved in anxiolytic and myorelaxant effects; the α_5_ subtype is largely expressed in the hippocampus and is associated with cognition processes like learning and memorising; the α_4_ and α_6_ subtypes, less investigated, are known as benzodiazepine-insensitive binding sites because they do not bind diazepam but recognize several non-benzodiazepine ligands.

Following the introduction in the clinical usage of chlordiazepoxide in 1960, a huge number of chemically heterogeneous classes of compounds have been reported in literature as BzR ligands [11]. This binding site is located at the α/γ subunits’ interface, distinct from the GABA binding sites situated at α/β interfaces (Figure 1). 

The pharmacological actions of BzR ligands range from full agonism (associated with anxiolytic, anticonvulsant, sedative-hypnotic, and myorelaxant effects) to antagonism (implying the ability to reverse sedation caused by agonists) and to inverse agonism (characterized by anxiogenic, somnolytic and proconvulsant effects) [12,13]. 

In 1987 Schofield and coworkers reported the sequence and the functional expression of the GABA_A_ channel [14]. Subsequently to this pioneering work, further knowledge about the subunits composing the GABA_A_ isoforms was achieved [15,16,17]. Since 2000, molecular genetics experiments and measurements of binding and efficacy of BzR ligands at each of the GABA_A_ isoforms [18,19,20,21] paved the way for the search of compounds provided by affinity- and/or efficacy-based selectivity for α_1_, α_2_/α_3_, α_5_ BzR subtypes [22].

Currently, the many BzR ligands available as drugs are agonists endowed with the above- mentioned depressant effects (ascribed to their action on the α_1_ subtype) and the antagonist flumazenil, employed as antidote to treat benzodiazepine overdose. Given the therapeutic potential of BzR ligands, this class of compounds has been one of the most intriguing and challenging field of medicinal chemistry research.

Our studies of indole derivatives as BzR ligands began in 1985 with the synthesis and the evaluation of the binding affinities of racemic *N*-(indol-3-ylglyoxylyl)amino acid derivatives (**1**) [23]. Compounds of series **1** were designed taking the 3-ethoxycarbonyl-β-carboline (**2**), reported to bind with nanomolar affinity to the BzR [24], as a reference structure.

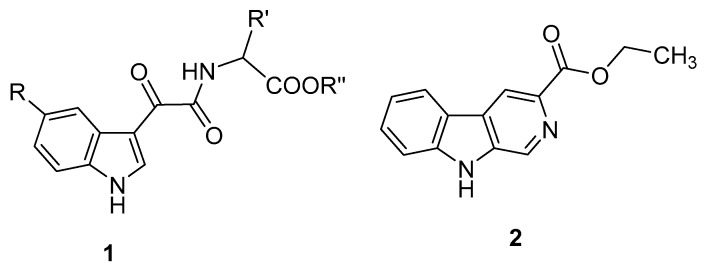



In series **1**, the groups at the 5 position of the indole nucleus (R) were H, Cl, Br, OCH_3_, NO_2_, while the R’ substituents of the aminoacid moiety were hydrogen, alkyl, benzyl and indolylmethyl, some of which bearing small groups on their benzene rings. R’’ was a hydrogen in the subset of carboxylic acids and a methyl or an ethyl in the subset of esters.

At that time, the endpoint in our biological experiments was the ability of the tested compound to displace a radioligand (generally [^3^H]flunitrazepam) from neuronal membranes obtained by the cortex of bovine brain. In the light of the knowledge about the GABA_A_ isoforms acquired several years after our initial studies, such binding data correlated mainly with the α_1_ BzR subtype.

Most of the compounds of series **1** exhibited affinity values in the micromolar-submillimolar range. The esters were more potent than the corresponding acids. In the ester subset two compounds were endowed by submicromolar affinity, both bearing a methyl group as R’, while the R substituent was a chlorine or a nitro group.

In a subsequent paper [25], we described the synthesis and the binding affinity for BzR of optically active forms of some *N*-(indol-3-ylglyoxylyl)aminoacids of series **1**. Expectedly, the esters performed better than the corresponding acids. The two compounds provided with the highest affinity as racemic mixtures in the previous work [23] did not show appreciable differences in affinity when tested as pure optical isomers, thus suggesting that the α-methyl side chain does not occupies a sterically hindered cleft within the BzR.

In 1992 we reported a series of *N*-(indol-3-ylglyoxylyl)-β-arylethylamines (**3**) as BzR ligands [26]. In this series, R was H, Cl, Br, NO_2_; R’ was H or CH_3_; the β-arylethyl side chain derived from tryptamine, tyramine, dopamine, α-phenylethlylamins bearing various substituents on the phenyl ring.

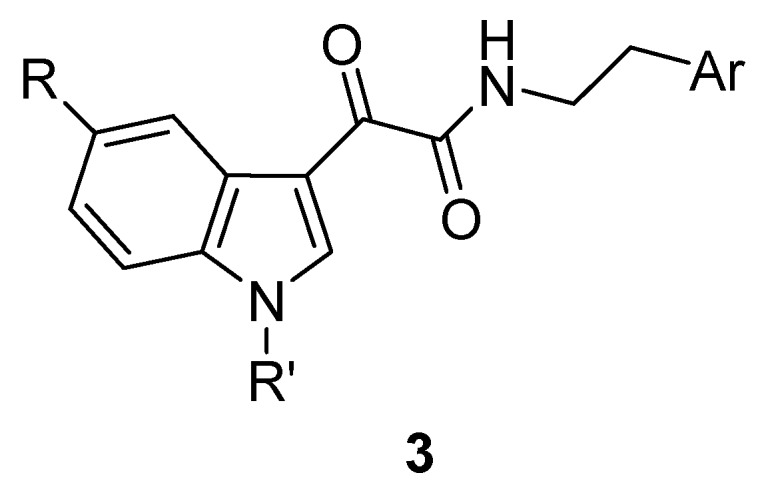



All the 1-methyl derivatives (R’ = CH_3_) were inactive, suggesting that the NH of the indole nucleus is engaged in a H-bond with the BzR or, alternatively, is sterically repelled by the binding site. The best ligands of the new series showed K_i_ values of 85 nM (R = H, R’ = H, Ar = *m*-methoxyphenyl) and 90 nM (R = H, R’ = H, Ar = *p*-methoxyphenyl). Surprisingly, when the hydrogen at the 5-position of the indole scaffold of the two above compounds was replaced by a nitro group affinity decreased to a considerable exent (K_i_ > 10 μM). The remaining compounds showed K_i_ values in the micromolar range.

In order to highlight the role of the indole NH fragment in the interaction with the BzR, we prepared a number of benzofurane and benzothiophene derivatives of general formula **4** and, respectively, **5** in which the above fragment was replaced by an oxygen or, respectively, a sulphur [27].

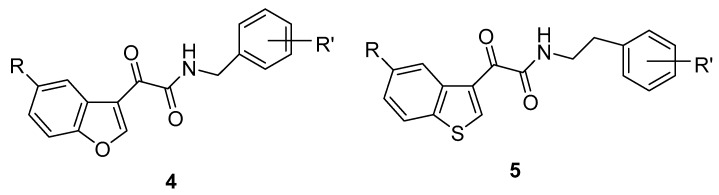



The new compounds were much worse in terms of potency compared with the corresponding isosteric indoles reported in our previous papers [23,26], clearly indicating that the indole NH donates a H-bond to an acceptor heteroatom of the BzR.

With the aim of finding indole derivatives with improved affinity for the BzR, three subsets of indolylglyoxylamides (general formulae **6**, **7** and **8**) were shortly after synthesized and tested [28]. In the three subsets R = H, Cl, NO_2_ while R’ = H, *p*-F, *p*-Cl, *p*-OCH_3_, *m*,*p*-(OCH_3_)_2_.

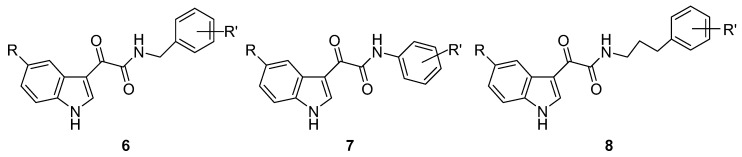



While the anilides **7** and the γ-phenylpropylamides **8** showed poor affinity, several benzylamides **6** displayed nanomolar potency. The scarce performance of indoles **7** and **8** was ascribed to steric repulsive interactions taking place between their side chain phenyl rings and the boundaries of the BzR. The excellent binding data of some benzylamides **6** suggested that their phenyl ring was correctly oriented to find room into a hydrophobic region of the BzR. The best performing ligand of series **6** (K_i_ 11 nM) had R = NO_2_ and R’ = *m*,*p*-(OCH_3_)_2_. The structure-affinity relationships in series **6** were characterized by interdependent effects of the R and R’ substituents on potency. Particularly, affinity of the 5-Cl/NO_2_ derivatives was improved by hydroxyl/methoxy substituents on the side chain phenyl ring, while affinity of the 5-H derivatives was increased by halogens on the same phenyl ring. Actually, such a pattern of interdependent effects of the substituents at the 5-position of the indole scaffold had already been observed in series **3**.

In order to identify indole derivatives as BzR ligands characterized by good water solubility, we prepared a number of *N*’-phenylindol-3-ylglyoxylhydrazides (**9**) [29]. In this series we inserted the following substituents on the parent structure: R = H, Cl, NO_2_ and R’ = H, *p*-F, *p*-Cl, *p*-NO_2_, *m*- or *p*-CH_3_, *p*-OH, *p*-OCH_3_.

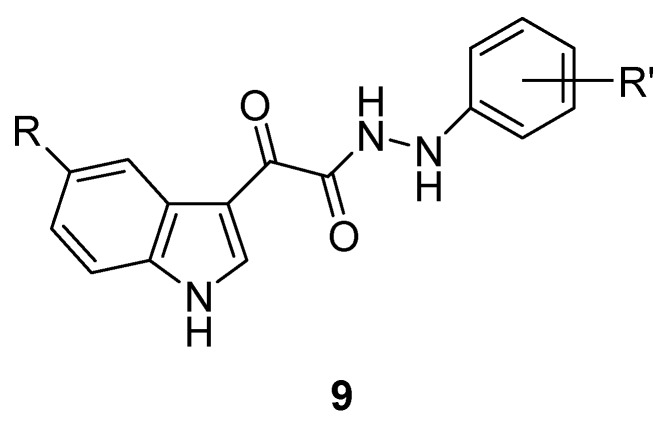



Affinity of compounds **9** was restricted to the 5-H derivatives, whereas the 5-Cl/NO_2_ derivatives were all devoid of affinity. Again, the structure-affinity relationships of these two subsets of compounds were divergent. In an attempt to explain the reasons underlying such findings, we searched the Cambridge Structural Database [30] looking for differences in the conformational properties of *N’*-arylhydrazides and *N*-arylamides. We realized that benzylamides **6** can adopt a transoid conformation about the C-N-C-Ar torsion angle (values comprised between −150° and +150°). Such a staggered conformation is forbidden to hydrazides **9** which are forced into a *gauche* disposition about the corresponding C-N-N-Ar torsion angle (values comprised between −60° and −120° or between +60° and +120°). 

**Figure 2:**
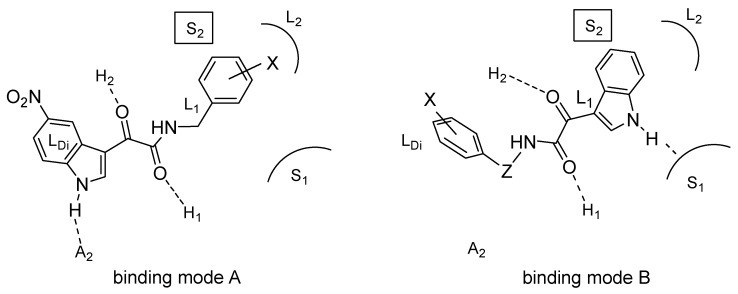
The binding modes A and B hypothesized for the 5-Cl/NO_2_ indoles and, respectively of the 5-H indoles oriented in the framework of the Cook’s pharmacophore/topological model [31]. Z is a CH_2_ in benzylamides **6** or a NH in hydrazides **9**.

The above data led us to hypothesize that our indole derivatives might bind to BzR by adopting one out of two conformations and orientations within the binding cavity depending on the substituent at the 5-position of the indole nucleus. On the basis of this conjecture, we began to consider the 5-Cl/NO_2_ and the 5-H indole derivatives as different “families” of BzR ligands, each displaying peculiar structure-affinity relationships. In our speculations we were aided by the pharmacophore/topological model proposed by Cook and coworkers [31] defined by the same authors as “comprehensive” because it holds for agonists, antagonists and inverse agonists. This model includes the following interaction sites: (i) a H-bond acceptor (A_2_), (ii) a H-bond donor (H_1_), (iii) a bifunctional H-bond donor/acceptor (H_2_/A_3_), and (iv) four lipophilic pockets (L_1_, L_2_, L_3_, and L_Di_). The boundaries of the binding site are defined in terms of sterically forbidden sites (S_1_, S_2_, and S_3_). Regarding the efficacy profile, the only safe statement was that filling of the L_3_ pocket (occupied by the diazepam pendant phenyl ring) was mandatory for agonism. The two putative binding modes (named A and B) of our indole derivatives are depicted in Figure 2 in the framework of Cook’s model. The binding mode A of the 5-Cl/NO_2_ indoles requires a staggered conformation of the side chain and gives rise to the following interactions: (i) the indole NH is H-bond to the A_2_ site; (ii) the C=O1 and C=O2 are H-bound to the H_2_ and H_1_ sites, respectively; (iii) the CH_2_, the phenyl and the fused benzene ring fill the L_1_, L_2_, and L_Di_ pockets, respectively. The binding mode B is accessible only to 5-H indoles because the sterically forbidden S_2_ site, closely facing the 5-position of the indole nucleus, cannot host substituents larger than a hydrogen. Such a binding mode, compatible with a folded conformation of the side chain, is characterized by the following interactions: (i) C=O1 and C=O2 are H-bound to the H_2_ and H_1_ sites, respectively; (ii) the lipophilic L_1_ and L_2_ pockets are filled by the pyrrole and, respectively, the benzene moieties of indole; (iii) the indole NH donates a H-bond to a heteroatom belonging to the S_1_ site. It is worth noticing that each of the two postulated binding modes benefits from three H-bonds with the BzR, consistently with the similar affinities displayed by the best performing 5-Cl/NO_2_ and 5-H indoles derivatives [29].

Some years later, we prepared and tested several *N*-(indol-3-ylglyoxyl)arylalkylamides **10**–**15** characterized by conformationally or geometrically constrained side chains, most of which featured a chiral center [32]. We reasoned that such properties of the new side chains might increase the chances of conferring affinity-based selectivity for any of the BzR subtypes.

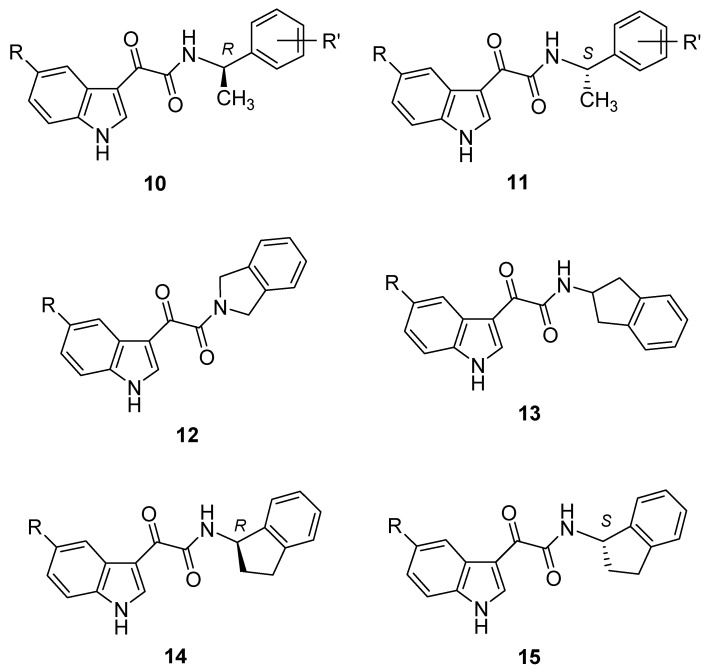



In series **10**–**15** the R substituents were H, Cl or NO_2_. In series **10** and **11** the R’ substituents on the phenyl ring were H, *p*-CH_3_, *p*-OCH*_3_*, *m*,*p*-(OCH_3_)_2_ and *p*-NO_2_. All of the *S* isomers (**11** and **15**) lacked affinity, likewise the optically inactive compounds **13**. Some of the *R* isomers displayed K_i_ values in the micromolar/nanomolar range. The structure-affinity relationships of the *R* isomers confirmed our hypothesis about the different binding modes of the 5-Cl/NO_2_ derivatives and of the 5-H derivatives. Specifically, in series **10** and **14** the 5-Cl/NO_2_ were significantly more potent than the 5-H derivatives. Conversely, in series **12** affinity was appreciable (K_i_ 123 nM) only if R = H. The most potent compound described in this study belonged to series **10** (R = NO_2_, R’ = H, K_i_ 17 nM). An overlay of molecular models of a few indole derivatives representative of the four series investigated showed that the inactive ones projected portions of their side chains into the sterically forbidden S_1_ subsite of Cook’s model. A subset of compounds were tested for their ability to displace [^3^H]flumazenil from recombinant rat α_1_, α_3_ and α_5_ BzR subtypes. All of them displayed high affinity for α_1_β_3_γ_2_ receptors and moderate to good selectivity for α_3_ and α_5_ subtypes. 

A number of *N*-(heteroarylmethyl)indol-3-ylglyoxylamides **16** were subsequently investigated [33] to probe the H-bonding properties previously ascribed to the S_1_ subsite of the BzR [29] (see binding mode B in Figure 2). The new compounds had R = H or NO_2_ at the 5-position of the indole moiety and several heterocycles on the side chain (Het).

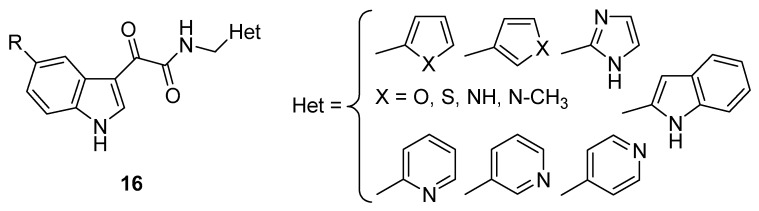



**Figure 3:**
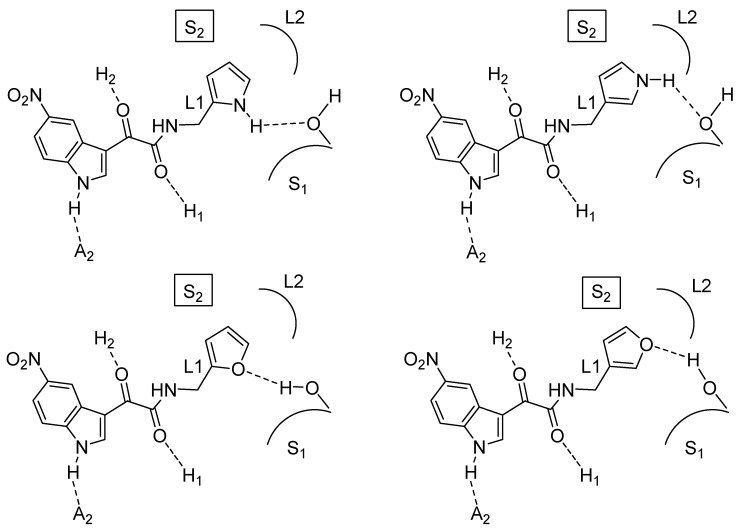
Putative binding mode A of some of the most potent ligands of series **16** hypothesized to interact with a hydroxy group located at the S_1_ subsite of the BzR.

The 5-NO_2_ derivatives bearing a 2-pyrrolyl, a 2-furyl, a 4-methyl-2-furyl, a 3-pyrrolyl or a 3-furyl ring in the side chain exhibited affinities in the nanomolar range (K_i_ values comprised between 13 nM and 33 nM) comparable with the most active compounds in the benzylamide series **6**. All the 5-NO_2_ derivatives bearing in the side chain a 2-thienyl, a 1-methyl-2-pyrrolyl, a 2-indolyl or a 2-imidazolyl were scarcely potent (K_i_ values in the micromolar range) or practically devoid of affinity. These data were explained as follows: the side chains of the most potent 5-NO_2_ indoles make H-bonds at the S_1_ site (Figure 3); the less potent or inactive NO_2_ indoles cannot make the same H-bonds if the heterocycle lacks a H-bond donor fragment (2-thienyl, 1-methy-2-pyrrolyl), is exceedingly bulky (2-indolyl) or is hydrophilic (2-imidazolyl).

All the 5-H derivatives of series **16** showed significant lower affinities (K_i_ in the submicromolar-micromolar range), probably because they are not able to engage H-bonds with their side chains in the binding mode B. 

Four of the most potent compounds of series **16** were tested for their ability to displace [^3^H]flumazenil from recombinant rat α_1_, α_2_ and α_5_ GABA_A_/BzR pure isoforms. All of them showed binding selectivity for the α_1_ subtype over the α_2_ and α_5_ subtypes.

In continuing our research of indole derivatives endowed with selective affinity for BzR subtypes, we came back to indol-3-ylglyoxylamides of series **6** by inserting lipophilic substituents at the 4′-position of the side phenyl ring, some of which were characterized by considerable steric bulk (i.e., Br, CH_3_, C_2_H_5_, C≡CH, C≡C-CH_3_, C≡CSi(CH_3_)_3_ and C≡C-CH_2_Si(CH_3_)_3_) [34]. In addition to the novel benzylamide derivatives, in the same paper we also disclosed indole derivatives in which the benzyl moiety was replaced by alkyl groups (i.e., (CH_2_)_3_CH_3_, (CH_2_)_4_CH_3_, CH(CH_3_)_2_, CH(CH_3_)CH_2_CH_3_, C(CH_3_)_3_ and CH_2_CH(CH_3_)_2_). Unfortunately, the above structural changes did not improve affinity for the BzR. One of the new compounds (R = NO_2_ and R’ = *p*-CH_3_) exhibited nanomolar potency at the rat recombinant α_1_ BzR subtype (K_i_ 31 nM) with no appreciable affinity for the α_2_ and the α_5_ subtypes, displaying a full agonist efficacy profile and a zolpidem-like sedative-hypnotic activity in vivo.

According to the Cook’s research group, the shapes of the BzR subtypes are very similar, with the exception of α_1_ and α_5_ subtypes that seem to be slightly larger at the lipophilic pockets, called L_Di_ and L_2_ regions, respectively [35]. The above steric differences have been exploited to obtain ligands that bind selectively to either α_1_ or α_5_ subtypes [22,36], but have also hampered the identification of affinity-based α_2_ and/or α_3_ selective ligands [37]. Investigators realized that a more fruitful strategy to obtain non-sedating anxiolytic agents or non-sedating cognition enhancers would be identifying compounds binding to all four subtypes, but preferentially activating only the targeted subtypes. The search of efficacy-based α_2_ and/or α_3_ selective ligands has indeed yielded better results [38]. Following this approach, we selected some of our indole derivatives of series **6**, **10** and **14** and tested their affinity and efficacy for the rat recombinant α_1_, α_2_ and α_5_ subtypes [39]. Efficacy was evaluated by measuring the ^36^Cl^−^ uptake in transfected human embryonic kidney cells stably expressing the three rat subtypes upon treatment with the tested compound. The results of this work led to the identification of two *N*-(indol-3-ylglyoxyl)benzylamides showing α_2_ selective efficacy in vitro and anxioselective effects in vivo according to the mouse light/dark box test [40], namely one from series **6** (R = H and R’ = *p*-F) and one from series **10** (R = NO_2_ and R’ = *p*-CH_3_). Docking calculations of the two anxioselective indole derivatives, using a homology-built model of α_1_ BzR provided by Cromer Bet al. [41], were carried out by means of 10 ns molecular dynamics. Although homology building approaches should regard with cautions, our in silico simulations showed that the selected 5-NO_2_ and the 5-H indole derivatives adopt two different orientations and conformations within the BzR, corresponding to mode A and, respectively, to mode B conjectured in our previous papers [29,32,33,34]. Most of the interactions (H-bonds, lipophilic pockets, sterically forbidden regions) observed in the docking complexes were in a reasonable agreement with those hypothesized using the Cook’s pharmacophore/topological model (Figure 2).

## 3. Indole Derivatives as Ligands of the Translocator Protein

In 1977 Braestrup and Squires discovered an alternative binding site for diazepam in rat kidneys and called it peripheral benzodiazepine receptor (PBR) to distinguish it from the BzR of the GABA_A_ ion channel [42]. Subsequently, wide studies on this protein allowed a deeper knowledge about its structure, tissue distribution and subcellular localization as well as physio-pathological functions. In virtue of these new findings, in 2006 Papadopoulos and collaborators proposed a new name for PBR, that is translocator protein (TSPO) [43]. 

TSPO is a transmembrane protein distributed in many tissues, mainly in those involved in steroid biosynthesis, including kidney, testis, liver and lung; in the CNS, it is mainly expressed in glial cells and, at lower levels, in neurons [43]. TSPO is located at the contact site between the inner and outer mitochondrial membrane, in strict association with other proteins that make up the mitochondrial permeability transition pore (MPTP): the voltage dependent anion channel, the adenine nucleotide transporter and the steroidogenesis regulatory protein. TSPO is involved in many biological processes, including mitochondrial respiration, cell proliferation and differentiation, induction of apoptosis. Moreover, TSPO plays a crucial role in steroid biosynthesis [44,45,46], specifically in the translocation of cholesterol from cytoplasm to inner mitochondria, which represents the rate-limiting step of steroidogenesis. As next step, cholesterol is converted by cytochrome P450 side chain cleavage (P450scc) into pregnenolone, the precursor of all steroid hormones [44,45,46].

The basal expression of TSPO is altered in different pathological conditions: an up-regulation occurs in brain injury and pathologies involving neuroinflammation (e.g., neurodegenerative diseases, gliomas) and in certain tumors; a down-regulation is often observed in correlation with anxiety disorders and post-traumatic stress [47,48,49]. In addition to cholesterol [50], TSPO binds with high affinity to structurally different synthetic compounds [51], such as Ro5−4864 (**17a**) [52] and PK11195 (**17b**) [53], both widely employed as reference ligands.

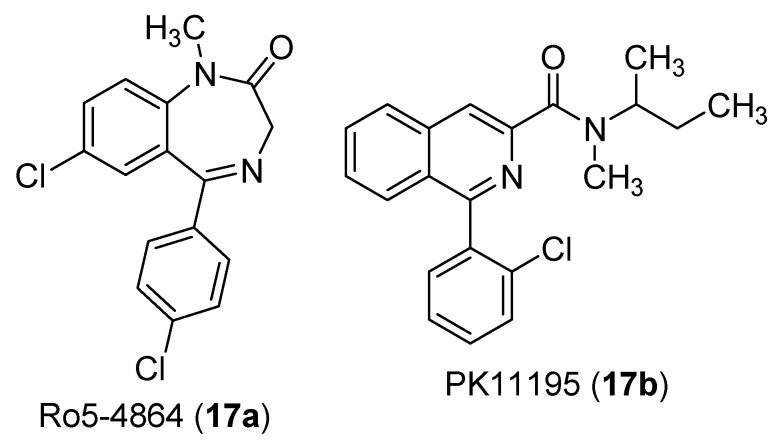



TSPO ligands may act as positive allosteric modulators of their target protein. Consequently, such ligands promote pregnenolone formation and increase the levels of endogenous steroids which, in turn, produce several physiological effects and result beneficial in pathological conditions of the CNS including inflammatory, neurological and psychiatric disorders [48,54]. In the last years, TSPO ligands are emerging as promising anxiolytics with favorable safety and tolerability and limited unwanted effects, like sedation, with respect to benzodiazepines [55,56,57]. Such pharmacological properties are ascribed to their ability to stimulate the synthesis of neurosteroids in the CNS, such as pregnenolone and allopregnanolone, which act as positive allosteric modulators of the GABA_A_ chloride channel [55,56,57]. Examples of these non-sedating anxiolytic agents are emapunil (**17c**) [58] and etifoxine (**17d**) [59], the latter dually binding to TSPO and to a site on the β-subunit of the GABA_A_ channel.

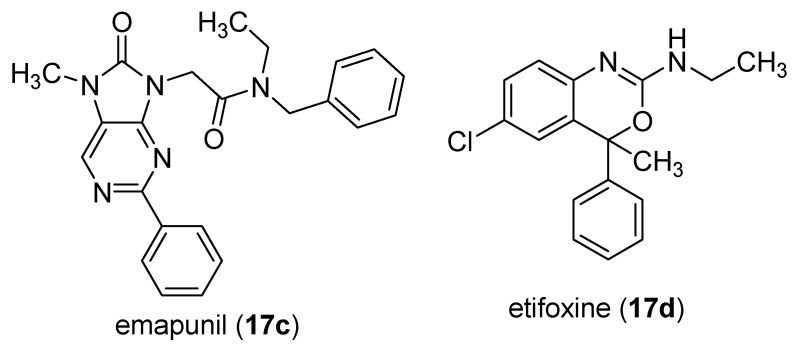



In 2004 and 2008 our research groups described the synthesis and the biological evaluation of a series of *N*,*N*-dialkyl-2-phenylindol-3-ylglyoxylamides (PIGAs, **18**) [60,61] designed as conformationally constrained analogues of 2-arylindoleacetamides **19** reported by Kozikowsky et al. as TSPO ligands [62]. In the general formula **18**, R_1_ and R_2_ were symmetric or asymmetric alkyl (linear and branched) or benzyl chains, R_3_ = H, CH_3_, F, Cl, NO_2_, CF_3_; R_4_ = H, OCH_3_, F, Cl, NO_2_; R_5_ = H, CH_3_, Cl.

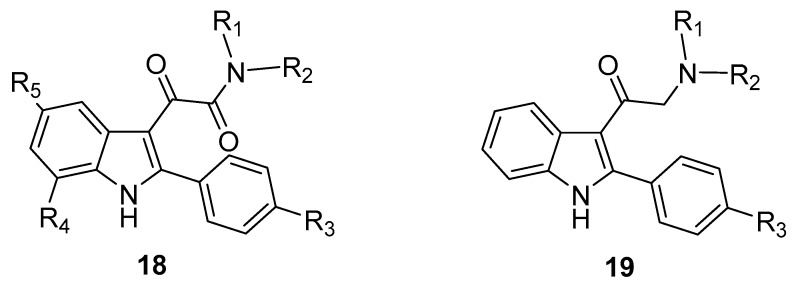



**Figure 4:**
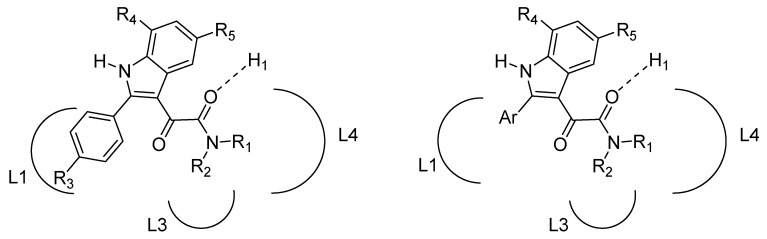
Pharmacophore/topological model of interaction between PIGAs and TSPO.

Most of the PIGAs showed affinity values for TSPO in the low nanomolar/subnanomolar range and full selectivity over the BzR. Noticeably, the most performant compounds exhibited a gain in affinity of one order of magnitude with respect to the indoleacetamide counterparts **19**. The structure-affinity relationships within this class were rationalized by means of a pharmacophore/topological model made by three lipophilic pockets (L1, L3 and L4, hosting the 2-susbtituted phenyl group and, respectively, the substituents R_1_, and R_2_ on the amide nitrogen) and a H-bond donor group H1 interacting with the amide carbonyl oxygen (Figure 4). 

PIGA ligands were further characterized for their efficacy measured as ability to improve the synthesis of pregnenolone in rat C6 glioma cells. A considerable number of them resulted stimulators of steroid biosynthesis more active than PK11195 and Ro5-4864 [60,61]. 

The best-performing PIGAs, in terms of affinity for TSPO and pregnenolone production, were evaluated in rats for their anxiolytic properties by means of the elevated plus-maze test [61,63,64]. In this assay, compounds **18a**,**b** exhibited non-sedating anxiolytic properties. Results from investigations about the mechanism of their anxiolytic activity indicated that it involves the stimulation of endogenous neurosteroid production, which in turn determines a positive modulation of the GABA_A_ chloride channel permeability [63].

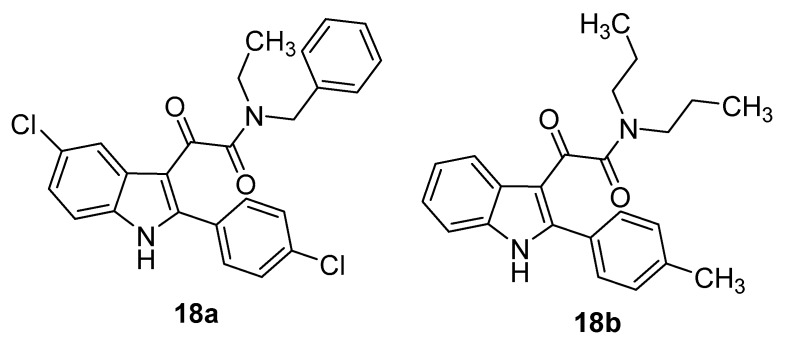



More recently, we investigated two novel series of PIGAs **18** featuring polar R_3_ groups (OH, NH_2_, COOH) on the 2-phenyl moiety or different 2-aryl substituents (Ar = 3-thienyl, *p*-biphenyl, 2-naphthyl) [65]. The 2-naphthyl derivatives exhibited the highest affinity values, thus confirming the crucial role of the ligand-receptor interaction involving the L1 pocket. In the same paper we reported a docking model of interaction between TSPO and three selected PIGAs based on a 3D structure of the target protein complexed to PK11195 [66,67]. This model was fairly consistent with the pharmacophore/topological scheme depicted in Figure 4, with the exception of the H1 site.

In two subsequent studies, a set of highly steroidogenic PIGA ligands demonstrated promising pharmacological activities [68,69]. Particularly, a number of these compounds were found to promote the oxidative metabolism of human astrocytes and prevent the oxidative damage and the inflammatory response in C6 glioma cells. The observed effects were completely counteracted by the co-treatment with D,L-aminoglutethimide, an inhibitor of P450scc involved in steroid biosynthesis, supporting the hypothesis that the PIGA-mediated protective mechanisms are mainly related to steroid production [68,69]. According to these results, PIGAs can be regarded as potential new therapeutic tools for the treatment of inflammatory-based neurodegenerative diseases characterized by astrocyte loss [68,69]. 

TSPO has been reported as a marker to reveal the onset of diseases related to its expression [47,49]. In virtue of the high affinity and selectivity of PIGAs for TSPO, we exploited the 2-phenylindol-3-ylglyoxylamide scaffold to develop specific TSPO molecular probes. In detail, reversible and irreversible fluorescent probes featuring the 7-nitrobenz-2-oxa-1,3-diazol-4-yl group (**18c**,**d**) were synthesized, characterized for their optical properties and tested in spectroscopy experiments to evaluate their ability to specifically label the mitochondrial localization of TSPO in *Drosophila* S2, rat C6 and human U87MG glioma cells [70,71,72]. These molecular probes emerged as useful tools to study the physiological role and the expressions levels of TSPO, especially the irreversible probes, whose lasting signal is maintained even after multiple washes, allowing a detection that is less affected by unspecific signal [71,72].

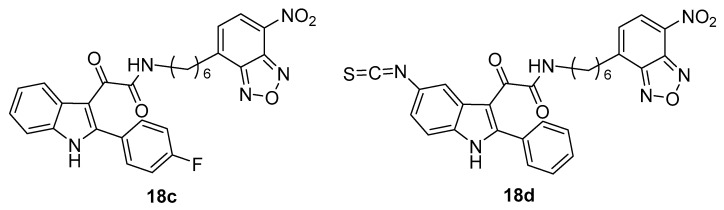



Shortly after, we synthesized *N*,*N*-di-*n*-propyl-(N1-[^11^C]methyl-2-(4′-nitrophenyl)indol-3-yl) glyoxylamide (**18e**) as a high affinity radiolabelled probe of TSPO [73]. The corresponding unlabeled compound was selected from a small library of PIGAs thanks to its optimal combination of high TSPO binding affinity and moderate lipophilicity (calculated logP = 3.9) to ensure adequate brain entry and low non-specific binding [73]. Compound **18e** was evaluated with positron emission tomography in monkey after being administered by intravenous injection. This probe readily entered monkey brain and gave a high proportion of specific and reversible TSPO binding, auguring well for its future application in humans [73].

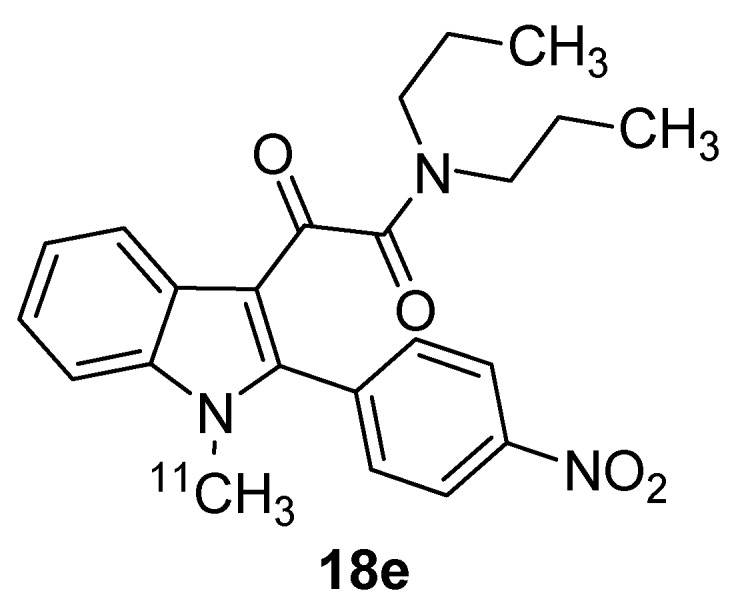



In the last years, our attention has focused on the lack of correlation between binding affinity and steroidogenic efficacy of TSPO ligands [74]. This represents a problem affecting the identification of effective lead compounds by a traditional affinity-based drug discovery strategy as well as the interpretation of pharmacological data [75]. Our efforts took advantage from studies showing that the biological effectiveness of a certain molecule cannot directly be deduced by its affinity for the target, but it may rather be related to the period for which it interacts with its target, defined as “residence time” (RT) [75]. This kinetic parameter corresponds to the reciprocal of the dissociation rate (K_off_) of the ligand-target complex. Based on these findings, we recently investigated whether RT could be employed to estimate the steroidogenic efficacy of a TSPO ligand. For this purpose, we selected a set of representative PIGAs showing different combinations of TSPO affinity and steroidogenic properties in vitro [76]. Then, a kinetic radioligand binding assay was set up with rat kidney membrane homogenates and validated for determination of RT values using [^3^H]PK11195. Our experiments showed a direct correlation between the efficacy of TSPO ligands and their RT values [76,77]. These findings were further supported by two studies in which we retrospectively assessed the relationship between RT and the steroidogenic activities of emapunil and etifoxine [78,79]. 

Subsequently, computational studies were performed to get insights into the different kinetics of PIGAs. Specifically, the unbinding paths of three representative compounds were studied by enhanced-sampling molecular dynamics simulations, revealing that subtle structural differences between PIGAs produce relevant effects on the unbinding energetics, involving mainly the LP1, TM2, and TM5 domains of TSPO. This study accounted for the molecular basis of the efficacy of TSPO ligands [80]. 

## 4. Indole Derivatives as Dual Ligands of the Translocator Protein and the Murine Double Minute 2 Protein

The pathogenesis of malignant gliomas involves the aberration of several signaling pathways, and a number of targets have been identified for therapeutic approaches, including growth factor ligands, receptors and intracellular downstream effectors [81]. As these deregulated intracellular signaling pathways are points of convergence from different stimuli, the concept of multi-target therapy is currently considered a rational approach to develop innovative and more efficient therapies [82,83,84].

In glioblastoma multiforme (GBM), a particularly aggressive form of brain malignancy, the p53 protein and TSPO, both acting as apoptosis inducers, represent two attractive intracellular targets [85]. Indeed, the loss of the ability of cells to undergo programmed cell death is a common step in cancer. A crucial step in the regulation of apoptosis is an increase of mitochondrial outer membrane permeability (mediated by the opening of the MPTP) and the release of specific transcription factors [86,87].

As reported in the previous chapter, TSPO is an important constitutive protein of the MPTP, holding a major regulatory significance in apoptosis [88]. Actually, a ligand from the class of PIGAs (**18**, R_1_ = R_2_ = *n*-butyl, R = Cl, R_4_ = Cl) had shown moderate antiproliferative and pro-apoptotic activity by enhancing the MPTP opening in rat C6 glioma cells [89].

The tumour suppressor protein p53 promotes apoptosis by interacting with members of the protective Bcl2-family proteins which in turn mediate the release of cytochrome c. P53 is a transcription factor that controls cellular response to stress by inducing cell cycle arrest or apoptosis [90,91]. The murine double minute 2 protein (MDM2) downregulates p53 activity by binding to the transactivation domain of p53. In response to stress, phosphorylation of p53 decreases the affinity of this protein for MDM2. A number of human tumors are associated with inhibition of the p53 pathway and consequent uncontrolled cell proliferation. Disruption of the p53-MDM2 interaction is therefore a therapeutic goal for the treatment of cancer [92]. The MDM2-p53 complex is stabilized mainly by a strong hydrophobic interaction between a region of MDM2 and the Phe19, Trp23, and Leu26 residues of p53. A synthetic molecule displaying three lipophilic groups in an orientation that mimics the presentation of the side chains of the above aminoacids can occupy the MDM2 cleft and thereby inhibit the p53-MDM2 interaction [92]. Based on these findings, computational methods were applied on our in-house library of indole-based TSPO ligands to identify those suitable to undergo appropriate decorations in order to inhibit the p53-MDM2 interaction and to maintain TSPO affinity. Following this approach, we synthesized a series of 2-phenylindol-3-yl-glyoxylamides (**20**), bearing on the glyoxylyl bridge a number of dipeptide moieties (L-Leu-L-Phe, L-Phe-L-Leu, L-Val-L-Leu, L-Leu-L-Val, L-Ile-L-Val, L-Ile-L-Ile and L-Val-L-Ile) capped as methyl or ethyl esters [93,94]. An immune−enzymatic assay on native human MDM2/p53 complex was performed to determine the ability (expressed as IC_50_ values) of the new compounds to bind MDM2 and disrupt the MDM2/p53 complex; affinity to TSPO was evaluated by competition binding assays employing the radioligand [^3^H]-PK11195 and expressed as K_i_ values. The strategy resulted successful as all the new compounds revealed to disrupt the p53-MDM2 complex and bind to TSPO at nanomolar concentrations.

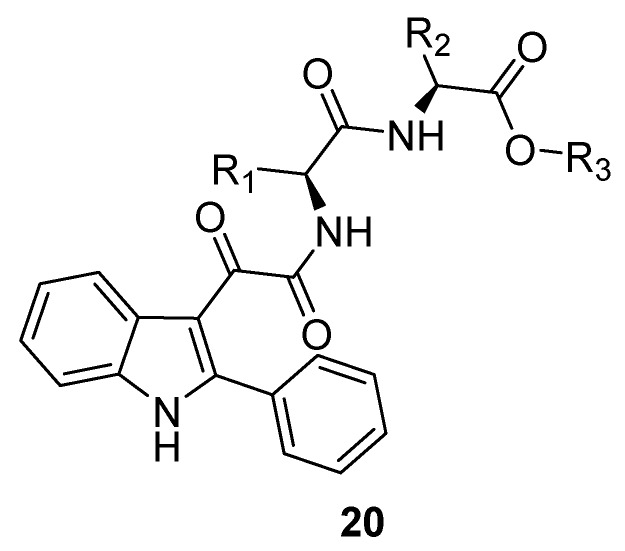



The compound from series **20** (R_1_ = CH_2_C_6_H_5_, R_2_ = CH_2_CH(CH_3_)_2_, R_3_ = CH_3_), showing the highest ability to dissociate the p53-MDM2 complex (IC_50_ = 4.3 nM) and the highest affinity for TSPO (K_i_ = 87 nM), was selected for further biological studies, giving the following results: (i) reactivation of the p53 function and inhibition of the GBM cell growth, triggering subsequent apoptosis; (ii) no efficacy on a GBM cell line expressing mutant p53, supporting the involvement of this protein in the observed effect; (iii) reduction of viability of glioma cancer stem cells (CSCs), which are less sensitive to anticancer agents and responsible for GBM recurrence [95]. These effects were significantly stronger than those elicited by the p53 activator nutlin-3 and the TSPO ligand PK11195 [91], thanks to the synergism resulting from the simultaneous activation of both targets [93,94]. Finally, cell viability assays performed on non-tumor human mesenchymal stem cells (MSCs) showed that the antiproliferative effect of the selected indole derivative was preferentially directed toward tumor cells. All these findings confirmed that dual targeting MDM2-p53 and TSPO is a valuable anticancer strategy against GBM, where the downstream p53 signaling is not mutated. 

Anticancer drugs binding reversibly to their targets may have several limitations in sustaining a therapeutic effect over time, thereby favoring the activation of alternative signaling pathways that escape drug action and cause resistance. Research in the field of oncology has recently been focused on the synthesis and development of new irreversible and long-lasting drugs [96]. As a continuation of our studies on 2-phenylindol-3-ylglyoxylyldipeptides **20**, we synthesized **21**, bearing a chemo-reactive isothiocyanate group at the 5-position of the indole nucleus. This compound, thanks to its ability to form covalent bonds with electrophilic groups, displayed a potent long-lasting binding affinity for TSPO and a prolonged inhibition of the MDM2-p53 complex [97]. Furthermore, **21** caused GBM cell death by arresting the cell cycle and inducing apoptosis; both effects were greater and more long-lasting than those of the reversible analogues of series **20**. The observed apoptotic effects were irreversible so that the cells were not able to regain proliferative activity after drug wash-out [97].

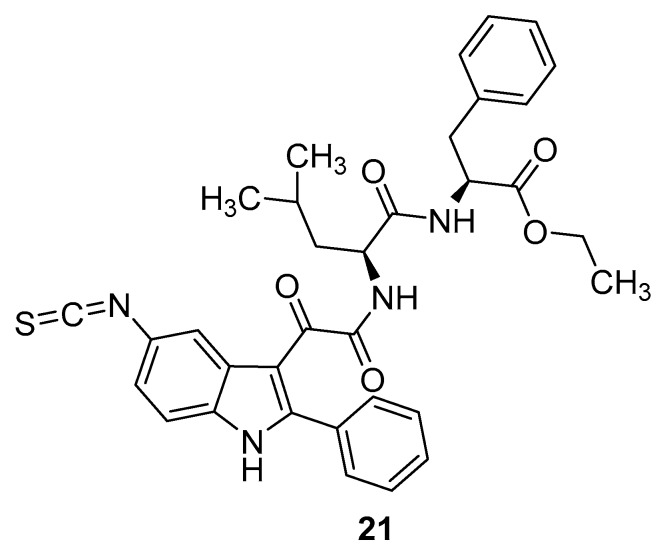



Compound **21** has been very recently employed in a study aimed to highlight the role played by the p53-MDM2 complex in osteoblast generation from MSCs [98]. The long-lasting MDM2-p53 dissociation determined by **21** enhances the MSC differentiation into osteoblasts through a pathway involving the G protein-coupled receptors kinase 2 and the A_2B_ adenosine receptor.

## 5. Indole Derivatives as Allosteric Modulators of the Human Adenosine A_2B_ Receptor

Adenosine plays a key role in a variety of physiological and pathological processes by interacting with specific receptors. Four different subclasses of adenosine receptors (ARs) have been identified to date, A_1_, A_2A_, A_2B_, and A_3_, all belonging to the superfamily of G-protein-coupled receptors [99,100]. Activation of ARs by adenosine or a synthetic agonist determines different intracellular events starting with inhibition (A_1_ and A_3_) or stimulation (A_2A_ and A_2B_) of adenylate cyclase. Additional molecular mechanisms coupled to occupation of ARs by agonists are stimulation of phospholipase C (A_1_, A_2B_, and A_3_), activation of potassium channels, and inhibition of calcium channels (A_1_) [101].

Being ubiquitously distributed in tissues and organs of mammalians, ARs have been considered attractive targets for the development of agonist- and antagonist-based therapies against a wide range of pathologies, including CNS disorders, cardiac arrhythmia, ischemic injuries, asthma, renal failure and inflammatory diseases [102].

In the course of our researches on BzR ligands (discussed in the second chapter), we prepared and tested a number of [1,2,4]triazino [4,3-*a*]benzimidazoles (TBI, **22**) as geometrically constrained analogues of indole derivatives (**23**) [103,104].

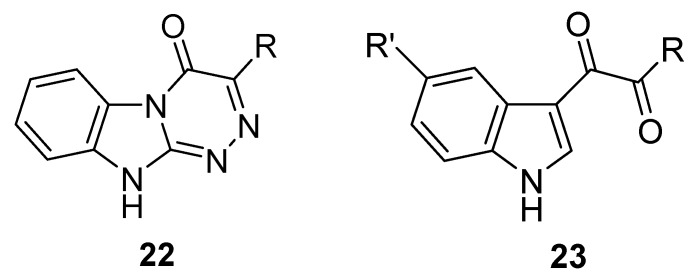



The most potent TBIs (R = C_6_H_5_, C_6_H_4_-*p*-OCH_3_, 2-furyl, 2-thienyl) displayed K_i_ values at the BzR (obtained from bovine cerebral cortex) ranging from 13 nM to 56 nM. 

It is worth noting that the structures of certain BzR ligands are similar to those of several antagonists of the A_1_ AR. Compare, as an example, the BzR agonist CGS-9895 (**24**) [105] with the triazoloquinazoline derivative CGS-15943 (**25**) identified as the first non-xantine antagonist of the A_1_, A_2A_ and A_3_ ARs [106].

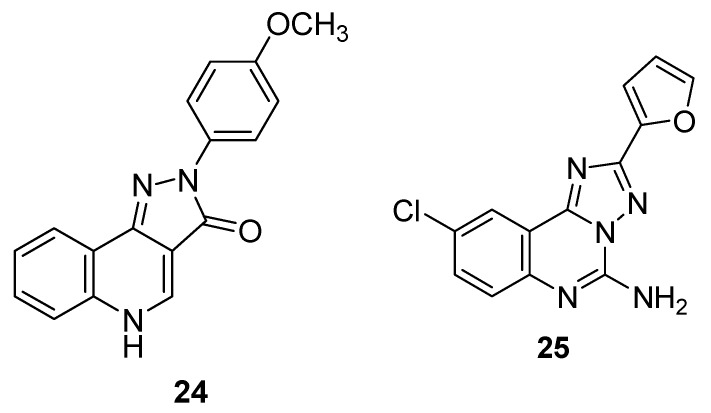



In the light of the above consideration, we prepared some novel TBIs (**26**) purposely designed as potential A_1_ AR antagonists through insertion of substituents not only at position 3 (R) but also at position 10 (R’) of the tricyclic system [107].

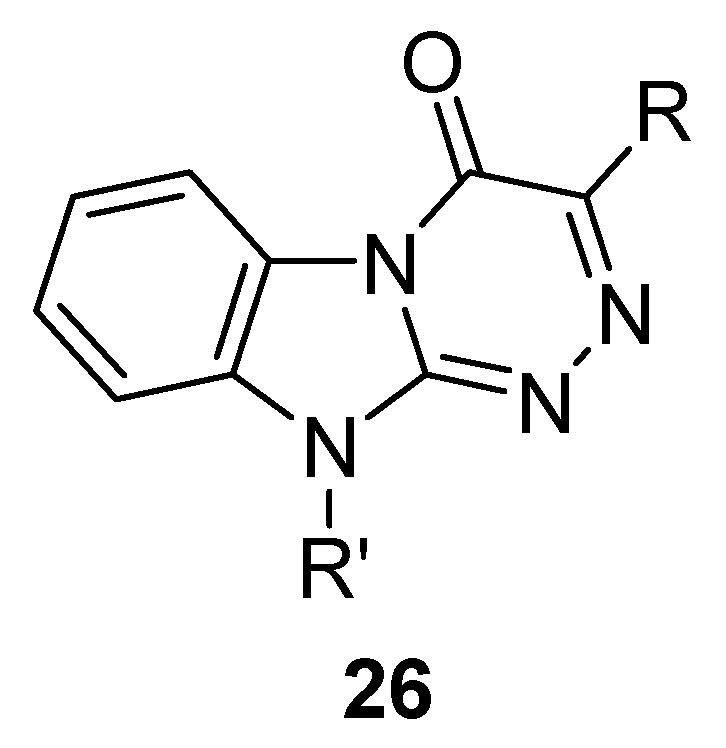



Among the new TBI derivatives, the most potent (K_i_ 83 nM) and selective one at the human A_1_ AR had R = R’ = phenyl [108].

In 2012 we disclosed a TBI derivative **26a** provided with high potency (IC_50_ of 3 nM) and selectivity for the human A_2B_ AR [109]. For a long time, this receptor has been less characterized compared with the other AR subtypes, partly due to the scarcity of specific ligands [110].

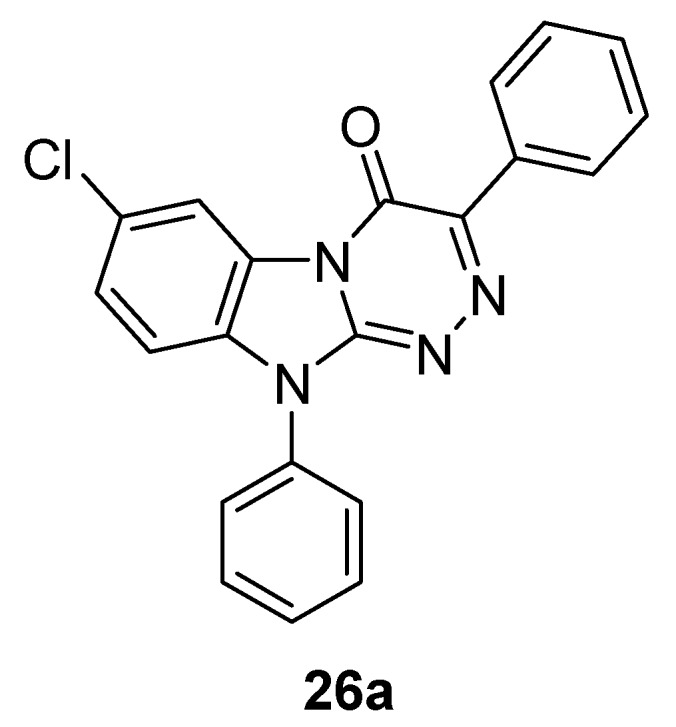



The therapeutic potential of agonists and antagonists of the A_2B_ AR is remarkable. Particularly, selective agonists of this receptor have been reported to reduce inflammation after ventilator-induced lung injury [111] and to modulate myocardial adaptation to ischemia [112]. Selective A_2B_ AR antagonists have been regarded as candidates for the treatment of cancer [113,114], colitis [115,116] and asthma [117,118,119].

Continuing our searches of novel lead compounds binding to ARs, we synthesized five indole derivatives **27a**–**c**, **28a**,**b** featuring a diketo moiety as a linker designed as open chain analogues of the TBI **26a**. Additionally, we purchased two indole derivatives **29a**,**b** characterized by an amide linker [120].

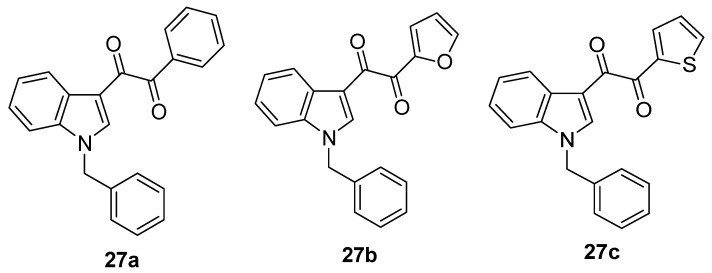

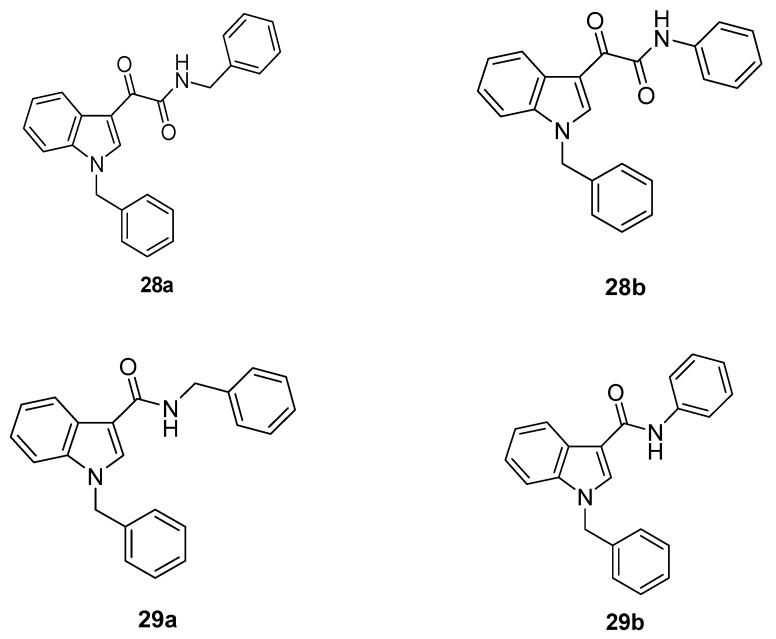





The affinity of indoles **27**–**29** for the human A_1_, A_2A_, and A_3_ AR expressed in CHO cells was determined by measuring their ability to displace specific radioligands from the above receptors. Compounds **27a** and **27b** exhibited submicromolar affinities for the A_1_ AR (K_i_ values of 161 nM and, respectively, 343 nM), whereas the remaining five indoles showed no appreciable affinity for the three AR subtypes. Functional experiments showed that **27a** and **27b** behaved at the A_1_ AR as antagonists. 

To evaluate the pharmacological effect resulting from interaction between **27**–**29** and the human A_2B_ AR, we measured to what extent our compounds modified the levels of cAMP in CHO expressing only this receptor. None of the compounds increased the cAMP levels at the concentration of 10 μM, clearly showing a lack of A_2B_ AR agonist activity. However, when the experiments were repeated in the presence of 5′-(*N*-ethylcarboxamido)adenosine (NECA), which acts as unspecific agonist of the ARs, **27a** and **28a**,**b** potentiated its agonist effects, suggesting that these three compounds interact with the A_2B_ AR as positive modulators. Conversely, **27b**,**c** and **29a**,**b** potently counteracted the NECA-mediated increase in cAMP, indicating that they act as negative modulators of the A_2B_ AR. 

We were very satisfied by these unexpected preliminary results (a typical case of serendipity) as our indole derivatives **27**–**29** are so far the only A_2B_ AR allosteric modulators reported in literature.

In a subsequent paper, the pharmacology of the new indole derivatives at the A_2B_ AR was characterized in more detail [121]. The potencies of compounds **27**–**29** in modulating the activity of A_2B_ AR agonists were determined by assessing the effects of different concentrations of each of them on cAMP accumulation induced by an EC_50_ concentration of NECA (100 nM). The resulting concentration-response curves indicated that **27a** and **28a**,**b** exhibit similar submicromolar potencies at A_2B_ AR, with EC_50_ values between 250 nM and 446 nM.

The concentration-response curves of **27b** and **29a**,**b**, obtained under the same conditions, fitted a two-site equation model, suggesting that these compounds recognize two sites of the A_2B_ AR with different affinities. The potency values obtained for the high and low affinity states of the receptor were in the subnanomolar/nanomolar and micromolar range, respectively. Conversely, the concentration-response curve of **27c** fitted a one-site equation model, revealing that this compound recognizes a unique site of the A_2B_ AR with nanomolar affinity.

Concentration-response curves in which the cAMP was measured by varying the concentration of the tested compound as well as the concentration of NECA gave us further information about their mechanisms of action. Particularly, from these curves we could infer that **27a** and **28a**,**b** enhance the efficacy of the agonist without affecting its potency, while **27b**,**c** and **29a**,**b** decrease either the efficacy and potency of the agonist. Several studies report that agonist efficacy and potency are not necessarily both modified by allosteric modulators [122,123]. A plausible hypothesis is that **27a** and **28a**,**b** affects specific conformational states of A_2B_ AR so as to improve the functional coupling to the intracellular signaling system without altering the conformation of the orthosteric site. Probably, **27b**,**c** and **29a**,**b** affects the agonist potency by shifting the receptor conformational states toward the resting ones; at the same time, they decrease the agonist affinity by deforming the conformation of the orthosteric site.

In virtue of their indirect mechanism of receptor modulation, allosteric modulators of G-protein-coupled receptors offer therapeutic advantages compared to agonists and antagonists. Particularly, they tune pharmacological responses only when and where the endogenous agonist is present in the specific tissue. Given the role played by A_2B_ AR in several physiological and pathological processes, discussed previously in this chapter, the positive and negative allosteric modulators of this receptor represent promising tools to identify novel druggable compounds.

Shortly after we reported the therapeutic potential of the indole derivatives acting as allosteric enhancers of A_2B_ AR agonists in the treatment of bone-related diseases (e.g., osteoporosis, rheumatoid arthritis, osteogenesis imperfecta, multiple myeloma, fracture mal-union) [124]. Particularly, we demonstrated that compound **28b** potentiates the effects of either adenosine and synthetic A_2B_ AR agonists in mediating osteoblast differentiation in vitro. In detail, by treating the MCSs with **28b** we observed an increase in the expression of osteoblast-related genes (runx2 and osterix) and osteoblast marker proteins (phosphatase alkaline and osteocalcin) associated with a stimulation of osteoblast mineralization.

## 6. Indole Derivatives as Ligands of the Kelch-like ECH-Associated Protein 1

Very recently, we have published a paper [125] disclosing novel indole derivatives binding to a pharmacological target playing a key role in cellular oxidative stress, namely the Kelch-like ECH-associated protein 1 (Keap1) [126]. Oxidative stress [127] is associated with an excess of reactive oxygen species (ROS), such as superoxide anion (O_2_^−^·), hydrogen peroxide (H_2_O_2_), hydroxyl radical (OH·). ROS are potentially cytotoxic as they damage DNA, RNA, enzymes and cellular membranes; they are generated from molecular oxygen during physiological processes (e.g., oxidative phosphorylation) or pathological events (e.g., inflammatory responses that protect our body from foreign pathogens). 

The cells reduce oxidative stress through radical scavengers (those best known are vitamins C, E and K) and antioxidant enzymes, both inactivating the ROS. The antioxidant enzymes include superoxide dismutase, catalase, heme oxygenase-1, glutathione S-transferase, NADPH:quinone oxidoreductase 1 and transketolase [128,129,130]. 

The expression of the above enzymes is regulated by the so called Keap1-Nrf2-ARE system [131], whose mechanism can be briefly schematized as follows. Under physiological conditions, the activity of the nuclear factor erythroid 2-related factor 2 (Nrf2) [132], a transcription factor, is inhibited by a strong interaction with Keap1. When the ROS exceed a safety threshold concentration, they disrupt the Keap1-Nrf2 complex by oxidizing a group of cysteine residues belonging to a specific domain of Keap1. This event triggers the release of Nrf2, allowing it to act as a transcriptional activator of genes that contain an enhancer sequence known as antioxidant response element (ARE) [133]. 

Inhibitors of the Keap1-Nrf2 interaction (KNI) are considered a promising new class of anti-inflammatory agents to treat diseases involving chronic oxidative stress, such as diabetes, cancer and neurodegenerative disorders [134]. 

The Keap1 binding cavity hosting the KNI inhibitors can be divided into six subpockets (P1-P6) [135]. P1 and P2 contain protonated arginine residues (Arg483, Arg415, Arg380), which give rise to strong electrostatic interactions with electron-rich parts of their ligands: salt bridges with carboxylate groups, H-bonds with nitro oxygens or azole nitrogens, cation-π contacts with aromatic rings. In the above mentioned six subpockets there are also lipophilic aminoacids.

Most of the KNI inhibitors reported in literature are chemically heterogeneous small molecules featuring a planar or quasi-planar scaffold which bear at least one aromatic ring and/or a weak acidic group involved in the abovementioned electrostatic interactions [136]. 

Our experience with indole as scaffold, led us to believe that it would be feasible to design indole derivatives acting as KNI inhibitors. The design of the compounds to be tested was mainly guided by the 3D structures of some KNI inhibitors co-crystallized with Keap1 [135,136,137]. With the help of molecular modelling and docking approaches, we selected nine indole derivatives **30a**–**i,** among which **30a**–**d** were synthesized, whereas **30e**–**i** were purchased.

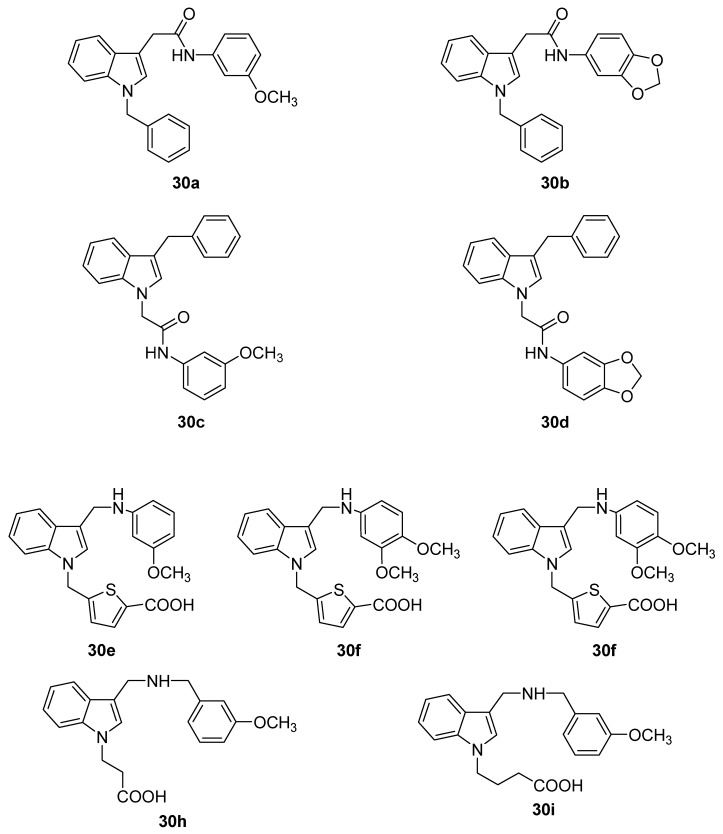



Based on their acid-basic properties, these compounds can be divided into three groups: (a) non-ionizable (**30a**–**d**); (b) acidic (**30e**,**f**); ampholytic (**30g**–**i**). The presence of methoxy group(s) or a methylendioxy moiety in the structures of **30a**–**i** was regarded as a chance for our compounds to strength potential cation-π interactions and/or engage H-bonds with the target protein. The thiophene ring featured by **30e**–**g** confers conformational rigidity and represents an electron-rich ring potentially able to interact with arginine residues. Compounds **30a**–**i** were evaluated for their ability to inhibit the Keap1-Nrf2 interaction through a cell-based luciferase reporter assay [138]. Nearly all of them were tested at the concentration of 10 μM; **30g** was tested at the concentration of 5 μM owing to its limited solubility in phosphate buffer. *t*-Butylhydroxyquinone (*t*-BHQ) was employed as a positive control [139] at the concentration of 50 μM, a value which gave in our experiments the maximum luciferase activity.

Compounds **30e**,**f** and **30g**, characterized by a 5-carboxythien-2-yl substituent, increased luciferase activity by 152%, 263% and 486%, respectively; their activities were higher by 3.2, 5.5 and 10-fold, respectively, than that exhibited by *t*-BHQ (48% increase). The remaining compounds displayed activities below 50%. A western blot analysis confirmed that **30e**,**f** and **30g** increase the expression of Nrf2 and of two enzymes encoded by its downstream target ARE genes, namely NADPH:quinone oxidoreductase 1 and transketolase. The same three best performing compounds showed to be non-cytotoxic when tested on human peripheral blood lymphocytes.

Docking simulations of the interaction between **30g** and Keap1, using available 3D structures of this protein [140], allowed us to explain the outstanding activity of this thiophene-contaning compound. The carboxylate group of **30g** makes a salt bridge with the Arg483 protonated side chain and a charge-reinforced H-bond with the Ser508 hydroxy group; the thiophene ring establishes a cation-π interaction with the Arg415 positively charged side chain. Such a cation-π interaction cannot be established by the less active compounds **30h**,**i** which bear a carboxylic group but lack an aromatic ring attached to the indole nitrogen. Furthermore, **30h**,**i** are much more flexible than **30e**,**f** and **30g**.

The (*m*-methoxy)benzylaminomethyl substituent of **30g** establishes hydrophobic interactions with Val512 and Leu472 side chains and a H-bond between the *m*-methoxy oxygen and the Leu472 backbone NH. The protonated nitrogen of **30g** is not involved in any type of electrostatic interaction. This suggests that the higher activity of **30g** with respect to those of **30e**,**f** may be ascribed to the different length of the linker between the indole nucleus and the *m*-methoxy moiety. The indole ring of **30g** contributes to the binding affinity through weak hydrophobic interactions with the Ala556 methyl group and the Arg415 dimethylene fragment.

## 7. Conclusions

The works described in this review confirm how useful and versatile indole can be as a molecular scaffold in designing drug-like molecules.
